# Schizophrenia-associated MicroRNA–Gene Interactions in the Dorsolateral Prefrontal Cortex

**DOI:** 10.1016/j.gpb.2019.10.003

**Published:** 2020-02-14

**Authors:** Danielle M. Santarelli, Adam P. Carroll, Heath M. Cairns, Paul A. Tooney, Murray J. Cairns

**Affiliations:** 1 School of Biomedical Sciences and Pharmacy, The University of Newcastle, Callaghan, NSW 2308, Australia; 2 Centre for Brain and Mental Health Research, Hunter Medical Research Institute, Newcastle, NSW 2305, Australia

**Keywords:** Schizophrenia, MicroRNA, Dorsolateral prefrontal cortex, Network, Neuropathology

## Abstract

**Schizophrenia**-associated anomalies in gene expression in postmortem brain can be attributed to a combination of genetic and environmental influences. Given the small effect size of common variants, it is likely that we may only see the combined impact of some of these at the pathway level in small postmortem studies. At the gene level, however, there may be more impact from common environmental exposures mediated by influential epigenomic modifiers, such as **microRNA** (miRNA). We hypothesise that dysregulation of miRNAs and their alteration of gene expression have significant implications in the pathophysiology of schizophrenia. In this study, we integrate changes in cortical gene and miRNA expression to identify regulatory interactions and **networks** associated with the disorder. Gene expression analysis in post-mortem prefrontal dorsolateral cortex (BA 46) (*n* = 74 matched pairs of schizophrenia, schizoaffective, and control samples) was integrated with miRNA expression in the same cohort to identify gene–miRNA regulatory networks. A significant gene–miRNA interaction network was identified, including miR-92a, miR-495, and miR-134, which converged with differentially expressed genes in pathways involved in neurodevelopment and oligodendrocyte function. The capacity for miRNA to directly regulate gene expression through respective binding sites in *BCL11A*, *PLP1*, and *SYT11* was also confirmed to support the biological relevance of this integrated network model. The observations in this study support the hypothesis that miRNA dysregulation is an important factor in the complex pathophysiology of schizophrenia.

## Introduction

Schizophrenia is a debilitating and complex psychotic disorder with various psychological and biological symptoms that result from a combination of genetic and environmental factors [1], [2]. Genetic studies have identified various susceptibility regions with modified genes of small effect that collectively contribute to the genetic predisposition of schizophrenia [3], [4], [5]. While many approaches have been used to elucidate the molecular mechanisms underlying the pathophysiology of the disorder, gene expression profiling studies in postmortem brain tissue have proven to be particularly complex and variable. Despite these challenges several differentially expressed schizophrenia candidate genes have been identified, including several involved in neurological, developmental, immune, and metabolic function [6], [7], [8], [9], [10], [11], [12], [13]. More recently the impact of common genetic variation associated with schizophrenia was examined in a very large postmortem tissue cohort established through the CommonMind consortium [14]. While many of these loci were observed to alter the expression of corresponding genes, even larger cohorts will be required to power the discovery of eQTLs for very small effect size variants. Expression studies more generally have been relatively inconsistent, and while discrepancy between study findings may be attributed to the heterogeneity there is some support for common biological pathways and processes, that result in the same phenotypic disturbances [3], [13], [14], [15], [16]. By utilizing Gene Set Enrichment Analysis (GSEA) approaches, the overall functional significance of individual gene changes can be integrated with a biological process or pathway despite heterogeneity amongst individuals [17].

As numerous pathways and biological functions are altered in schizophrenia, it is important to consider the observed changes in epigenomic influence of microRNA (miRNA; reviewed in [18]), these small non-coding molecules are capable of coregulating expression levels of large sets of target genes. They therefore have the capacity to direct regulatory convergence on specific biological pathways that can be attributed to the pathophysiology of this disorder. This potential was highlighted in a previous study [19], which alluded to the significance of gene–miRNA regulatory networks in the pathophysiology of schizophrenia in correlation analysis of miRNA target predictions. They also suggested that transcription factor binding sites within these miRNA genes produced feed-forward loops for complex patterns of cortical gene regulation. Similarly, a complex regulatory network model for cortical miRNA influence in schizophrenia is supported by genome-wide association implicating *MIR137* and a number of miR-137 target genes [20], [21]. With many predicted miR-137 targets also enriched among genes with sub-threshold significance, this suggests a potential role for this miRNA in regulating a key gene set associated with the disorder [22].

In the current study, we sought to further investigate potential gene-level pathways, functional disturbances, and gene–miRNA regulatory networks in schizophrenia by simultaneously performing high-throughput miRNA and mRNA expression analysis in the dorsolateral prefrontal cortex (DLPFC BA46) in a cohort of schizophrenia and non-psychiatric controls. This enabled us to directly explore the functional significance of altered miRNA on its target molecules and determine the biological significance at the pathway level using GSEA. The expression–correlation between miRNA–gene target pairs was found to be useful for revealing significant interaction networks relevant to the neuropathology of schizophrenia.

## Results

### Postmortem analysis revealed schizophrenia-associated changes in gene expression

**Figure 1:**
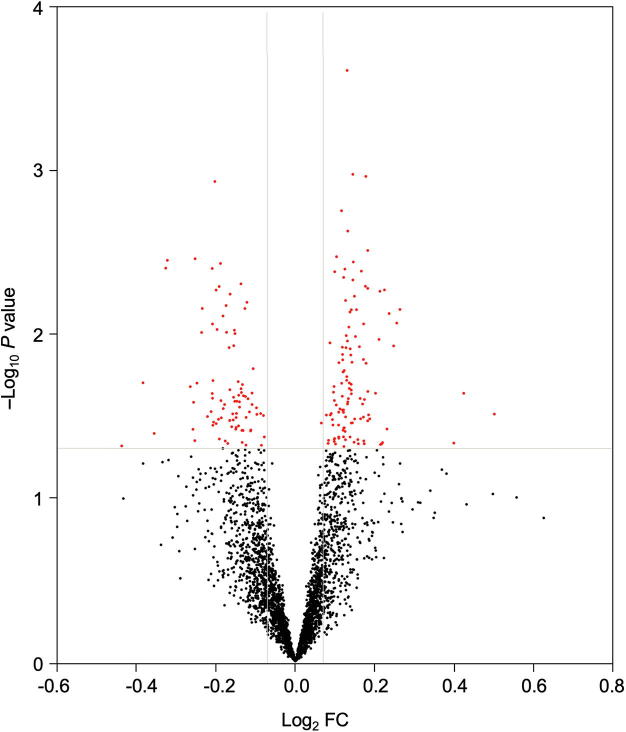
**Volcano plot of gene expression in DLPFC (BA46) of schizophrenia versus non-psychiatric controls** The significance of DEGs (−Log *P* value) was plotted against their corresponding Log_2_FC (disease/control) for 37 matched pairs of schizophrenia and non-psychiatric controls. Differential expression was determined using Bayesian empirical linear model (adjusted *P* < 0.05; FC > 10%). Downregulated genes are shown in top left quadrant and upregulated genes are shown in the top right quadrant with differentially expressed genes (DEGs) highlighted in red. All DEGs are listed in Table S1 with the associated FC and adjusted *P* values (Benjamini–Hochberg correction). DEG, differentially expressed gene; FC, fold change; DLPFC, dorsolateral prefrontal cortex.

**Figure 2:**
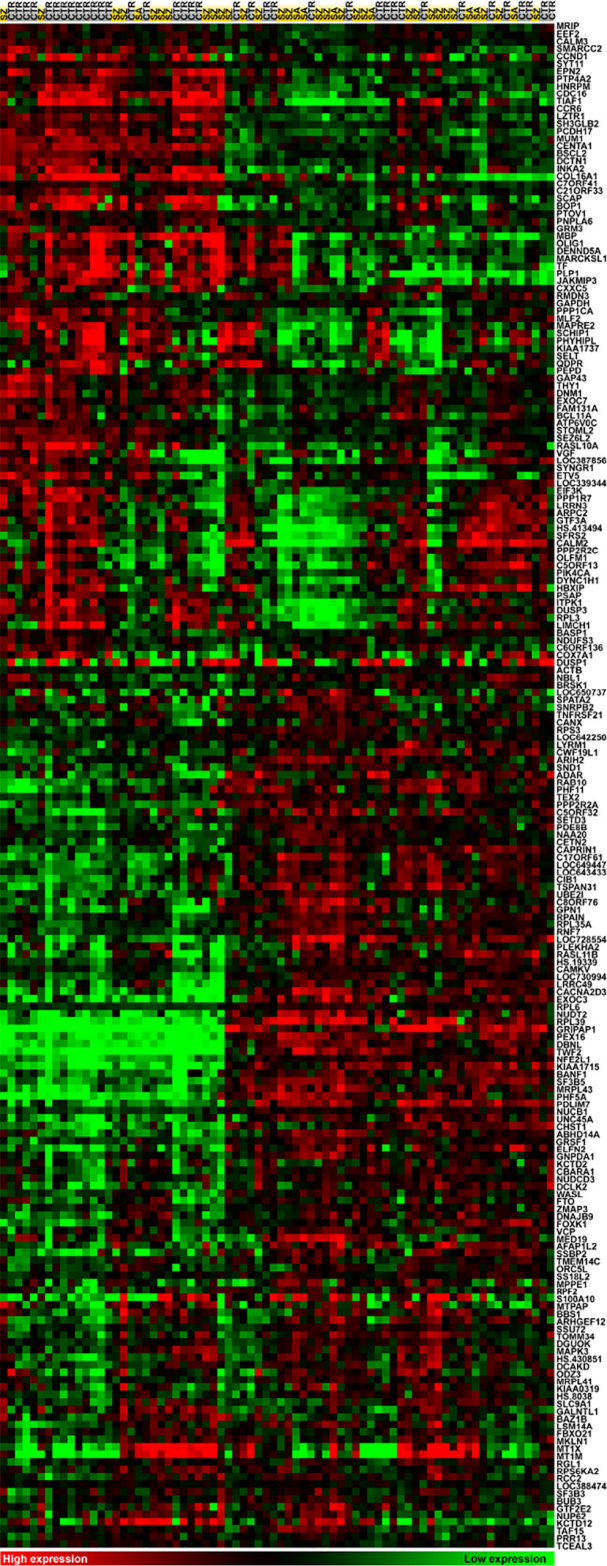
**Gene expression profiling in DLPFC (BA46)** Hierarchical clustering of DEGs identified (log transformed, median centered and uncentered correlation, average linkage clustering; Cluster 3.0) [45]. Gene expression levels are color coded with green for low expression and red for high expression. Heat map was generated with Java Treeview 1.1.1 [46]. Genes are listed on the right and designated by gene symbol, and subjects are listed on the top with cases in yellow and CTR in gray. There are two microarray probes corresponding to two different alternatively-spliced variants of *CANX*, therefore *CANX* was reported as DEG twice. These two variants were collapsed into one feature in Treeview due to their similar expression profiles. SZ, schizophrenia; SA, schizoaffective; CTR, control.

To explore the postmortem gene expression profile associated with schizophrenia we compared cases with matched controls from a non-psychiatric comparison group. A total of 205 differentially expressed genes (DEGs; FC   10% and adjusted *P* < 0.05) were identified with an almost even split of downregulated and upregulated genes using a Bayesian empirical linear model (Figure 1). A 10% FC threshold was applied to filter noise while allowing detection of modest phenotype-associated expression changes. Several schizophrenia candidate and neurologically relevant genes were differentially expressed, with downregulated genes including glutamate receptor metabotropic 3 (*GRM3*), calmodulin 2 & 3 (*CALM2/3*), proteolipid protein 1 (*PLP1*), quinoid dihydropteridine reductase (*QDPR*), synaptogyrin 1 (*SYNGR1*), synaptotagmin 11 (*SYT11*), oligodendrocyte transcription factor 1 (*OLIG1*), transferrin (*TF*), growth associated protein 43 (*GAP43*), and VGF nerve growth factor inducible (downregulated); and upregulated genes including myelin basic protein (*MBP*). Refer to Table S1 for the full list of DEGs. Unsupervised hierarchical clustering of DEGs was illustrated using a heat map to highlight clusters of genes with similar expression patterns with respect to cases and controls (Figure 2).

### Annotation of DEGs was enriched in neurological pathways

Functional annotation of DEGs and pathways was performed using the core analysis feature in IPA. This revealed enrichment of several schizophrenia-associated and neurological terms (Table 1; full results in Table S4). Interestingly, *dopamine receptor signaling* (*P* = 1.42E−03) was the second most enriched canonical pathway, and also enriched was *Dopamine-DARPP32 feedback in cAMP signaling* (*P* = 3.16 × 10^−3^). Significant association with schizophrenia was also reported (*P* = 4.74 × 10^−2^) with 11 associated genes, as well as first-onset paranoid schizophrenia (*P* = 2.17 × 10^−2^) (see full IPA results – Table S4). Other relevant enriched neurological functions and pathways included *synaptic long-term potentiation* (*P* = 5.37 × 10^−3^) and *long-term depression of neurons* (*P* = 5.21 × 10^−3^). Gene set enrichment analysis using GAGE also reported enrichment of several schizophrenia relevant pathways (Table 2), such as the neurodevelopmental signaling pathways *ErbB* and *Wnt signaling*, and processes such as *long-term potentiation*, *focal adhesion*, and *axon guidance*. Gene sets associated with Alzheimer’s and Parkinson’s disease were also observed to be enriched.

**Table 1:** **Top functional annotations of DEGs in DLPFC in schizophrenia**

*Note*: Summary of enriched categories identified by IPA core analysis. For the canonical pathways, both the number of DEGs and the number of all genes in the pathway are provided and expressed as the ratio.

**Table 2:** **Enriched KEGG pathways for DEGs in DLPFC in schizophrenia**

*Note*: Enriched KEGG pathways were identified by GAGE analysis (*P* ≤ 0.01). Ratio indicates the number of DEGs / the number of genes in the respective KEGG pathway. Pathways of interest to the study of schizophrenia are presented in bold.

### miRNA–mRNA interaction modules were enriched with terms associated with schizophrenia

**Figure 3:**
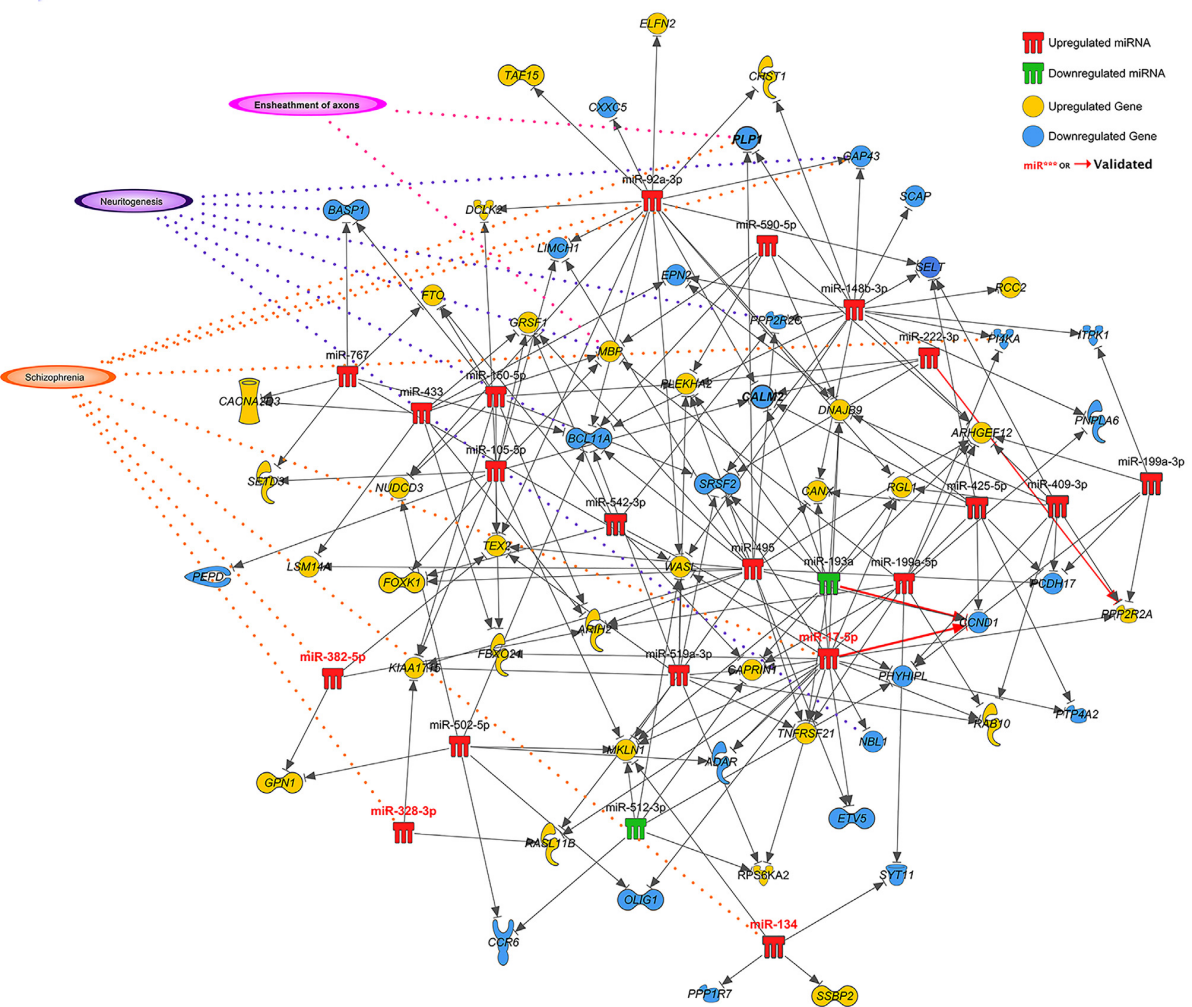
**miRNA–gene regulatory interaction network involving miRNAs and genes dysregulated in DLPFC** Associations with schizophrenia, neuritogenesis, and/or ensheathment of axons are indicated. Dysregulated miRNAs (miR***) and genes validated by qPCR are highlighted in red label or bold font. Red arrows indicate gene–miRNA interactions that have been experimentally observed according to Ingenuity Knowledge Base or luciferase reporter gene assays conducted in this study. The image is produced using the pathway designer tool in IPA.

**Figure 4:**
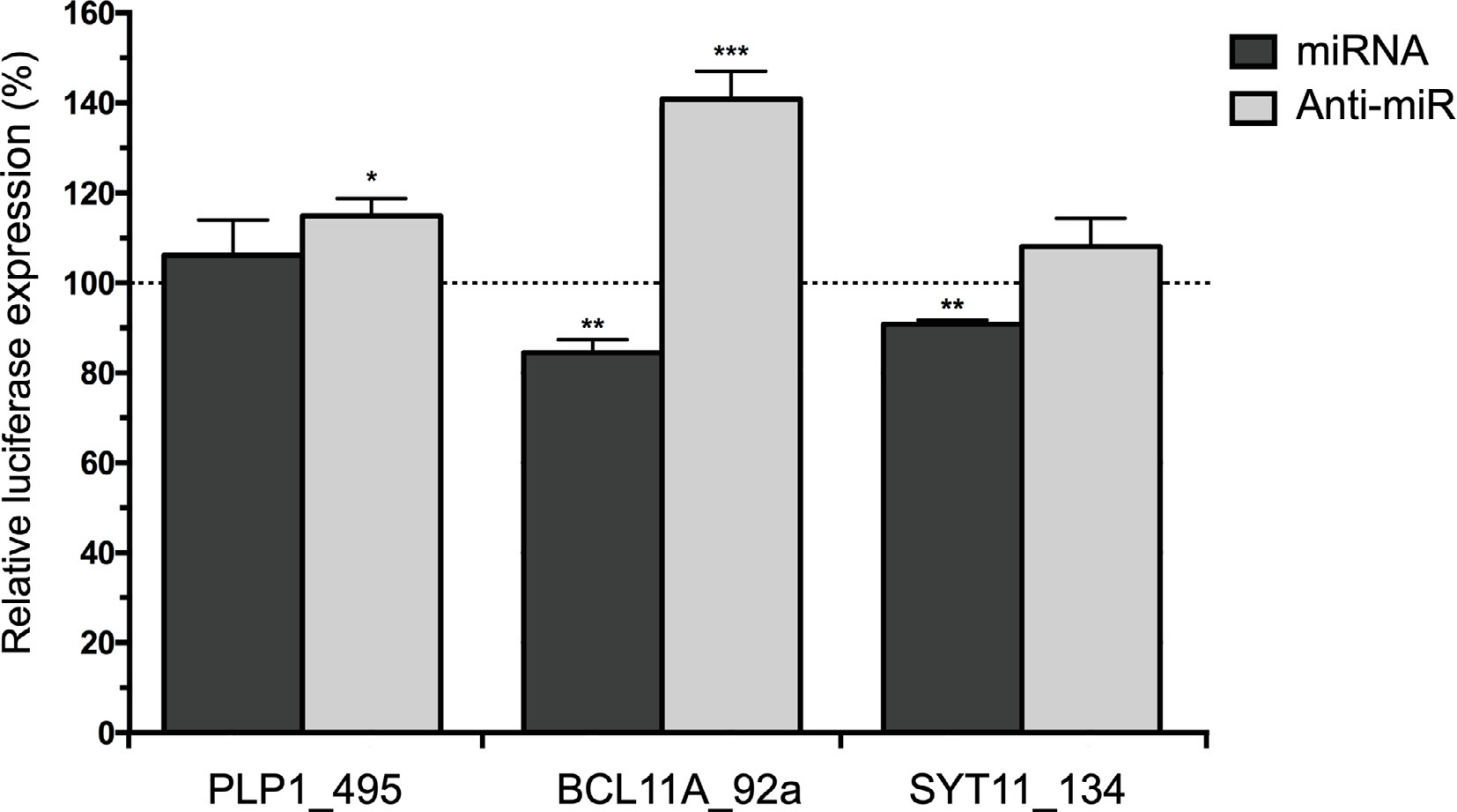
**Biological validation of schizophrenia-associated miRNA–gene interactions** Luciferase expression is presented as a percentage of firefly luciferase gene activity in response to synthetic miRNA mimic or inhibitor (anti-miR) relative to the renilla luciferase gene activity. Data are presented as average ± SD. *, *P* < 0.05; **, *P* < 0.01; ***, *P* < 0.001 (one tail student *t*-test).

To investigate posttranscriptional regulatory interactions affecting DEGs, we paired our cortical gene expression data with miRNA expression (Table S2), determined previously on the same samples, using the miRNA target filter analysis tool in IPA. This analysis reported 270 interactions (Table S3), involving 104 individual genes. A large proportion of these interactions (110), were high confidence predictions, including a number of experimentally supported pairing, previous observed in the literature including miR-17-5p and cyclin D1 (*CCND1*), miR-193b-3p and *CCND1*, and miR-222-3p and protein phosphatase 2 regulatory subunit B, alpha (*PPP2R2A*). The functional implications of these interactions were explored using the core analysis tool in IPA (Table 3; full results in Table S5). The majority of neurological and schizophrenia relevant pathways and functions that were reported in the standalone DEG functional analysis remained in the results of this miRNA interaction filter analysis, including *dopamine receptor signaling*, while *recycling of synaptic vesicles* (*P* = 6.14 × 10^−3^), *ensheathment of axons* (*P* = 1.11 × 10^−4^), and *synaptic transmission* (*P* = 2.12 × 10^−2^) were also enriched (see Table S5 for the full list of pathways). A network map of those experimentally observed or high confidence predicted interactions highlights associations with *schizophrenia*, *neuritogenesis*, and *axon ensheathment* (Figure 3). We validated three key negative regulatory interactions at key hubs in this network within an appropriate biological context by conducting luciferase reporter gene assays (Figure 4). Significant downregulation of reporter gene expression through the miR-92a binding site in *BCL11A* was observed upon increased miR-92a expression (15.5% decrease; *P* = 0.0053), while repression by this site by endogenous miR-92a was also de-repressed (40.9% increase; *P* = 0.0006) with an antisense oligonucleotide to inhibit endogenous miR-92a function. Significant downregulation through the *SYT11* binding site upon increased miR-134 expression was also observed (9.2% decrease; *P* = 0.0081), whilst there was only a trend for upregulation with the miR-134 inhibitor (8.1% increase; *P* = 0.1529 not significant). Transfection with miR-485 inhibitor, however, induced significant upregulation through the apparent de-repression of its cognate binding site in *PLP1* (14.9% increase; *P* = 0.0192).

**Table 3:** **Top functional annotations of miRNA-mRNA interactions in DLPFC in schizophrenia**

*Note*: Summary of enriched categories identified by IPA core analysis - microRNA target filter. For the canonical pathways, both the number of DEGs and the number of all genes in the respective pathway are provided and expressed as the ratio.

## Discussion

### Downregulated genes are functionally related to the neurobiology of schizophrenia

The neuropathology of schizophrenia is complex and heterogeneous, involving multiple layers of genetic and epigenetic dysregulation. High throughput analysis of gene expression provides important insight into the combination of these influences and the systems they effect. In the current study we observed both up and downregulated genes associated with schizophrenia, and while these may all have an impact on cortical function, the downregulated genes had the most obvious connection with biological processes known to be dysregulated in the syndrome (Table S1). This suggests the existence of a negative regulatory influence on neurologically sensitive genes and gene pathways in the DLPFC of subjects with schizophrenia. This observation is supported by our previous observations of schizophrenia-associated upregulation of miRNA in the same cohort [23] and other similar miRNA studies in schizophrenia (reviewed in [18]).

Interestingly, we observed downregulation of myelin-related and oligodendrocyte genes *PLP1*, *TF*, *OLIG1*, and *GAP43* — a class that is consistently reported as downregulated in schizophrenia across various brain regions [13], [24], [25], [26], [27]. *GAP43* is known for its involvement in neurodevelopmental synaptic plasticity and long-term potentiation [28] and has been previously observed as downregulated in DLPFC [29]. We also identified upregulation of *MBP*, which is also associated with oligodendrocyte function. *SYNGR1*, observed to be downregulated in schizophrenia, is located in the 22q13 region with reported functional mutations [30] and roles in synaptic plasticity and neurotransmission [31]. Downregulation of *SYNGR1* has also been reported previously in the PFC in schizophrenia [7]. *SYT11*, located in the 1q21-22 susceptibility locus and localized in synaptic vesicles [32] was also downregulated, along with *DUSP1* – previously shown to be downregulated in BA46 by Iwamoto and colleagues [33]. Downregulation was also observed for B-cell CLL/lymphoma 11A (*BCL11A*), a highly brain-expressed mediator of axon and dendrite arborization [34], and interestingly the closely related *BCL11B* has also been reported to be downregulated in peripheral blood mononuclear cells from patients with schizophrenia [35].

### Gene set analysis reveals enrichment of neurologically relevant pathways

The advantage of gene expression approaches to investigating polygenic disorders such as schizophrenia lies in the second tier of analysis that provides insights into potentially affected pathways and functions downstream of any combination of reported dysregulated genes. Convergence of these DEGs via functional pathways analysis was observed using Ingenuity Systems, reporting highly relevant terms to neurodevelopment, including neurological diseases such as schizophrenia and Parkinson-dementia syndrome, as well as dopamine receptor signaling. In addition, we used cut-off free gene set enrichment analysis (GAGE) to evaluate the distribution of gene expression within biological pathways. This approach allows redundancy at the individual gene level to accommodate for the heterogeneity that occurs between individuals within both the schizophrenia and control groups within the matched cohort. Using this approach, we observed disruptions in several KEGG pathways associated with oligodendrocyte function, and neurodevelopmental processes, including *cell adhesion, signal transduction, axonogenesis, neurotransmission, long term potentiation*, with the related disorders of *Parkinson’s* and *Alzheimer’s disease* were also identified. This is consistent with a number of other postmortem cortical expression studies in schizophrenia [4], [6], [7], [8], [9], [12].

### mRNA–miRNA regulatory networks are dysregulated in schizophrenia

In this study, we were particularly interested in identifying regulatory modules and networks defined by miRNA–mRNA interactions that may be associated with schizophrenia. We were in a position to do this as the miRNA expression was examined by microarray in the same samples [23]. In this miRNA study, we used predicted gene targets to develop an *in silico* model of the functional consequences of miRNA dysregulation using pathways analysis. This highlighted the potential for miRNA influence on axon guidance, long term potentiation, synaptic transmission, neurogenesis, and brain development, amongst many other schizophrenia and neurological pathways and functions. While these results were fascinating and relevant to the disorder, miRNA predictions can be prone to false positive and false negative attribution [36]. Their significance is also highly context specific as their transcriptional substrates will vary substantially between different tissue and cell types [37]. In the current study, we had the opportunity to integrate miRNA associated changes in the post-transcriptional regulatory environment directly with changes in target mRNA transcription using IPA. This enabled an investigation into the hypothesis that changes in cortical gene and miRNA expression in schizophrenia are intertwined into regulatory modules. We identified a regulatory interaction network that has significant association with schizophrenia and neurological processes. The validity of this network is supported by results of luciferase reporter gene assays conducted, in which negative regulatory interactions were successfully supported for miR-92a and *BCL11A*, miR-134 and *SYT11*, and *PLP1* and miR-495. These miRNA and gene interactions were selected for validation as they were suspected to be important network hub and node interactions respectively, with biological significance to the pathophysiology of schizophrenia.

The regulatory network consists of several schizophrenia candidate genes such as *SYT11*, *GAP43*, *OLIG1*, and *PLP*, along with miRNA such as miR-92a, miR-134, and miR-495. Once again, we also see enrichment of oligodendrocyte and myelination associated genes and functions in this regulatory network. Adding to this observation are several studies that also support a role for miRNA in oligodendrocyte differentiation, Letzen et al. [38] observed differential expression of several miRNAs with predicted target genes enriched for oligodendrocyte function and myelination in human embryo stem cells undergoing differentiation into oligodendrocytes. Our studies collectively support observations of decreased glial cell populations in schizophrenia brain (reviewed in [39]) and suggest implications for miRNA dysregulation in the cause of this schizophrenia-associated cytoarchitectural anomaly. This network is also enriched for genes involved in neuritogenesis, with all but one of these genes downregulated. This is especially interesting as localization of DICER to the somatodendritic compartment of neurons has been reported [40], and in our previous investigation in BA46 [23] we reported an upregulation of DICER expression. Dendrite formation requires an intricate coordination of gene expression, thus implicating a role for a miRNA regulation network, such that abnormalities to this network would have serious consequences to neural connectivity. This is especially fitting with the disconnection hypothesis of schizophrenia, which describes abnormal connectivity [41]. Interestingly, in this context, this network is also associated with *dopamine receptor signaling* and *dopamine-DARPP32 feedback in cAMP signaling* pathways. The disconnection hypothesis describes a model of abnormal neural circuitry via disturbances to neurotransmitter systems as well as connectivity [41]. Prior to our investigation there has been little work considering gene–miRNA interaction in schizophrenia. An earlier study by Sun et al. [42] explored the hypothesis that the many varying small effects that cause schizophrenia are organized into pathways and gene networks specific to the disorder. Guo et al. [19] identified regulatory networks of miRNA, gene targets and transcription factors by a computational meta-analysis in which target predictions of experimentally validated, schizophrenia-associated miRNA were correlated with schizophrenia candidate genes.

## Conclusion

**Figure 5:**
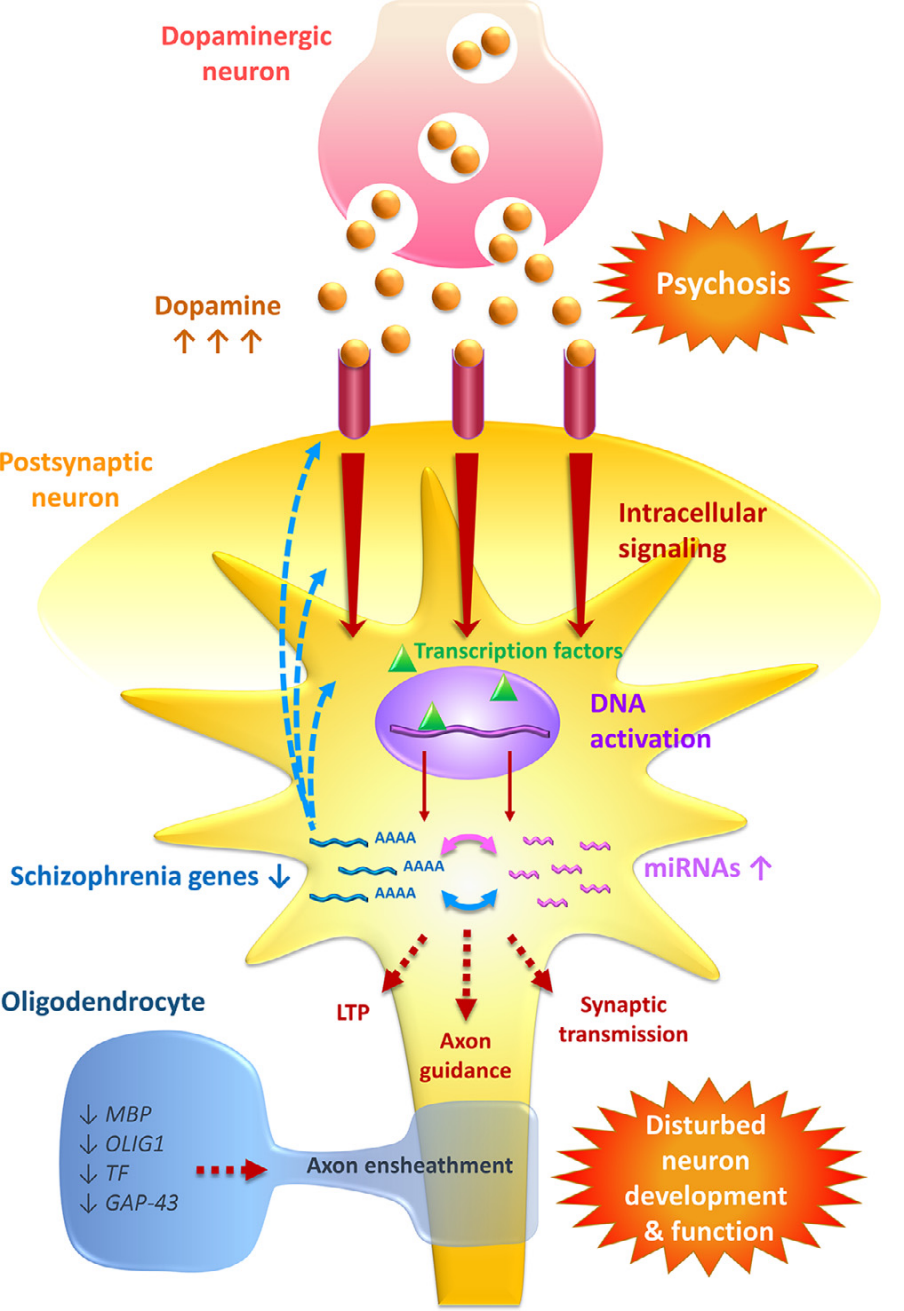
**A model for the role of miRNA–gene regulatory interaction networks in neurons in schizophrenia**Using the dopamine hypothesis as a starting point, dopamine (orange balls) receptor binding (pink tubes) on the postsynaptic neuron is enhanced and is associated with psychosis. The enhanced dopamine receptor binding would lead to increased intracellular signaling and DNA activation by transcription factors (denote by green triangles). We have observed upregulation of miRNA (pink) and downregulation of schizophrenia genes (blue) and a significant interaction network. Dysregulation of genes potentially affects upstream pathways and functions, such as dopamine receptor signaling, and downstream pathways and functions such as LTP, axon guidance, and synaptic transmission. We have also observed downregulation of oligodendrocyte genes, which has the potential to decrease axon ensheathment. The end result is a disturbance to neuron development and function, all consistent with the disconnection hypothesis. LTP, long term potentiation; MBP, myelin basic protein; OLIG1, oligodendrocyte transcription factor 1; TF, transferrin; GAP43, growth associated protein 43.

Our study expands on our understanding of the neuropathology of schizophrenia by incorporating differentially expressed predicted gene targets of differentially expressed miRNAs to imply gene–miRNA regulatory networks. In previous work we investigated changes in miRNA expression after antipsychotic drug treatment in wild-type mouse brain, and observed a gene–miRNA interaction network significant to both schizophrenia and mechanisms of antipsychotic function which also suggests a role for these molecules in the neuroleptic mechanism by way of multiplex gene regulation [43]. Interestingly, miRNA may also be playing a role in treatment resistance [44]. We explored gene–miRNA regulatory networks in peripheral blood mononuclear cells (PBMCs) from patients with schizophrenia and discovered important regulatory nodes of posttranscriptional regulation related to schizophrenia, particularly among downregulated genes [45]. More recently, we also observed a significant alteration of circular RNA (circRNA) in postmortem DLPFC, which are capable of participating in a competing endogenous RNA (ceRNA) network through the abrogation of schizophrenia-associated miRNA function through miRNA sponging [46]. The identification of these regulatory networks, with significant links to schizophrenia and other relevant neurological processes, strengthens the hypothesis that miRNAs and their extended posttranscriptional regulatory networks are highly plausible causal factors for the changes in gene expression seen in schizophrenia brain, and represent significant players in the pathophysiology of the disorder. To graphically illustrate the potential for miRNA–gene interaction networks to influence brain function in schizophrenia and their implications for the pathophysiology of the disorder, we designed a schematic which summarizes this hypothesis (Figure 5).

## Materials and methods

### Brain tissue cohort, tissue dissection, and RNA extraction

Postmortem brain tissues from BA46 of the DLPFC from 37 matched pairs of schizophrenia and schizoaffective disorder subjects and non-psychiatric controls were provided by the New South Wales Tissue Resource Centre (University of Sydney). Research application of human tissue was conducted in accordance with strict guidelines approved by the University of Newcastle Human Research Ethics Committee with written informed consent obtained from the next of kin. Cases with schizophrenia were diagnosed according to the Diagnostic and Statistical Manual of Mental Disorders-IV criteria and confirmed by medical file review with respect to the Item Group Checklist of the Schedules for Clinical Assessment in Neuropsychiatry. Full cohort description, including age, gender, matching, and analysis, is detailed in Table S6 and described previously by Weickert and colleagues [47]. Demographic variables [age, *P* = 0.95; pH, *P* = 0.49; postmortem interval (PMI), *P* = 0.23; and RNA integrity number (RIN), *P* = 0.81] do not differ between schizophrenia and control cohorts (two-tailed Student’s *t*-test). Tissue dissection was performed as previously described and RNA extracted using TRIzol reagent (ThermoFisher, Waltham, MA; catalog No. 15596026) [23], [47].

BA46 was chosen for examination as this area has been consistently linked to impairments to the monitoring of working memory, as is seen in schizophrenia patients [48]. Over-activation of BA46 during working memory tasks has also been reported in first-degree relatives of patients with schizophrenia [49].

### Gene and miRNA expression analysis

Gene expression profiling was conducted using the Sentrix HumanHT-12 Expression BeadChip platform developed by Illumina (version 3). For gene expression, TRIZol extracted total RNA was purified for use on the microarrays using the RNeasy MinElute Cleanup Kit (Catalog No. 74204) in accordance with the manufacturer’s instructions (Qiagen, Hilden, Germany. Purified RNA (500 ng) was amplified for hybridization to the BeadChips using the Illumina TotalPrep RNA Amplification Kit, and 750 ng of the resulting purified biotinylated cRNA was hybridized to the arrays using the Whole-Genome Gene Expression with IntelliHyb Seal Kit according to manufacturer’s instructions (Illumina, San Diego, CA). Microarrays were scanned using an Illumina BeadArray reader before background subtraction, variance stabilizing transformation and normalization using the robust spline method. Data was then uploaded to ArrayExpress to make it freely available for public download using the accession number E-MTAB-8386 (https://www.ebi.ac.uk/arrayexpress/experiments/E-MTAB-8386). DEGs were investigated using a Bayesian empirical linear model, implemented using the Linear Models for Microarray Data (LIMMA) package [50]. Benjamini–Hochberg correction for multiple comparisons was performed using the false discovery method (FDR). Hierarchical clustering of gene expression was performed using log transformed, median centered and uncentered correlation, average linkage clustering using the Cluster 3.0 package [51]. Heat maps were generated with Java Treeview 1.1.1 [52].

miRNA expression profiling was conducted previously on the same samples using the commercial microarray platform (Illumina) – total RNA preparation, amplification and hybridization were performed in accordance with the manufacturer’s instructions, as described previously [23].

### Gene pathways and functional analysis

The functional implications of the DEGs were explored using the core analysis feature within the Ingenuity Pathways Analysis (IPA) software (QIAGEN Inc., https://www.qiagenbio-informatics.com/products/ingenuity-pathway-analysis); *p*-values calculated in IPA are derived from Fisher’s exact text and are corrected for multiple testing using the Benjamini Hochberg false discovery method. Gene set enrichment analysis was performed on gene expression data using Generally Applicable Gene Set Enrichment (GAGE) (http://bioconductor.org/packages/release/bioc/html/gage.html) which uses a false discovery rate (FDR <0.05) to adjust for multiple testing [53].

### Integration analysis of DEGs and miRNA

Differentially expressed miRNAs from the previous investigation in Santarelli et al. [20] and mRNAs from the current investigation were expression-paired using the miRNA target filter tool in IPA. This tool pairs the DEGs that are predicted or experimentally observed targets of differentially expressed miRNAs by fold change, and target predictions are compiled using TargetScan [54], TarBase [55], miRecords [56], and the Ingenuity Knowledge Base. Experimentally-observed interactions and those of high or moderate confidence correlation were collected. The functional implications of the inferred interactions were explored using the core analysis feature within IPA and an interaction network mapped using the pathway designer.

### miRNA target gene reporter assay

Validation of miRNA–gene interactions within a biological system was achieved using a dual luciferase reporter assay procedure established in our laboratory and detailed in Carroll and colleagues [57]. Briefly, target gene 3′UTR constructs containing the miRNA recognition element (MRE) and *Spe*I and *Hin*dIII restriction overhangs were ligated into *Spe*I and *Hin*dIII cleaved pMIR-REPORT firefly Luciferase miRNA Expression Reporter Vector (ThermoFisher/Ambion; Catalogue No. AM5795) by the forced cloning method. Ligated vector was grown in DH5α chemically competent *Escherichia coli* cells and screened for success by Ampicillin selection and by sequencing extracted construct DNA. HEK-293 cells were cultured at 37 °C with 5% CO_2_ and 90% humidity in DMEM with 10% (vol/vol) fetal calf serum, 20 mM HEPES, 0.15% (wt/vol) sodium bicarbonate and 2 mM l-glutamine. Cells were seeded into 96-well plates at 4 × 10^4^ cell/well, and co-transfected using Lipofectamine 2000 (ThermoFisher; Catalogue No. 11668019) with the recombinant firefly luciferase reporter vector, an internal control renilla luciferase vector (pRL-TK), and either a commercial synthetic miRNA mimic (30 nM) or inhibitor (100 nM) (*mir*Vana™ ThermoFisher; Catalogue Nos. 4464066 and 4464084). The ratio of firefly: renilla luciferase expression is measured for each mimic or inhibitor against its respective *mir*Vana negative control to determine the success of gene inhibition (ThermoFisher; catalog Nos. 4464058 and 4464079). Each interaction was assayed four times, each performed in triplicate, and data analysed using a student’s *t*-test.

## Data availability

Raw data have been deposited into the ArrayExpress (ArrayExpress: E-MTAB-8386).

## Authors' contributions

MC designed the study, supervised the analysis and co-wrote the manuscript. DS wrote the manuscript and performed all the expression analysis and qPCR validation. AC performed reporter gene assays and contributed to manuscript preparation. HC contributed to manuscript preparation. PT assisted with the sample collection, study design, and critical appraisal of the manuscript. All authors have read and approve the final manuscript.

## Competing interests

The authors declare no competing interests.
