# Supplementary material for: Schizophrenia-associated MicroRNA–Gene Interactions in the Dorsolateral Prefrontal Cortex
Source: Genomics Proteomics Bioinformatics. 2020 Feb 14;17(6):623–34. doi: 10.1016/j.gpb.2019.10.003 (PMC7212302; doi:10.1016/j.gpb.2019.10.003)
Supplement: Supplementary data 2 [file mmc2.docx]

**Table S2 Differentially expressed microRNA in DLFPC (BA46) in schizophrenia**

| **Name** | **Expression**  **(normalised intensity)** | **FC** | ***q* value (%)** | ***P* value (qPCR)** | **Altered expression previously reported** | **PMID** |
| --- | --- | --- | --- | --- | --- | --- |
| hsa-miR-502 | [412](http://genome-www4.stanford.edu/cgi-bin/SMD/source/sourceResult?choice=Gene&option=Name&criteria=412.339508944075) | 2.76 | 3.65 |  | STG | 19721432 |
| hsa-miR-452-3p | [374](http://genome-www4.stanford.edu/cgi-bin/SMD/source/sourceResult?choice=Gene&option=Name&criteria=373.998502582476) | 1.67 | 0 |  | STG | 19721432 |
| hsa-miR-590 | [467](http://genome-www4.stanford.edu/cgi-bin/SMD/source/sourceResult?choice=Gene&option=Name&criteria=466.944330698385) | 1.60 | 0 |  |  |  |
| hsa-miR-519c | [451](http://genome-www4.stanford.edu/cgi-bin/SMD/source/sourceResult?choice=Gene&option=Name&criteria=450.887841516545) | 1.51 | 3.65 |  |  |  |
| **hsa-miR-382** | [1226](http://genome-www4.stanford.edu/cgi-bin/SMD/source/sourceResult?choice=Gene&option=Name&criteria=1225.70142706245) | 1.47 | 0 | 0.0434 |  |  |
| hsa-miR-548b | [392](http://genome-www4.stanford.edu/cgi-bin/SMD/source/sourceResult?choice=Gene&option=Name&criteria=391.863450029098) | 1.44 | 0 |  |  |  |
| **hsa-miR-652** | [2515](http://genome-www4.stanford.edu/cgi-bin/SMD/source/sourceResult?choice=Gene&option=Name&criteria=2514.8455309291) | 1.38 | 0 | 0.0322 | PBMC | 21738743 |
| hsa-miR-433 | [3255](http://genome-www4.stanford.edu/cgi-bin/SMD/source/sourceResult?choice=Gene&option=Name&criteria=3255.4603529429) | 1.35 | 0 |  |  |  |
| hsa-miR-592 | [8353](http://genome-www4.stanford.edu/cgi-bin/SMD/source/sourceResult?choice=Gene&option=Name&criteria=8353.47059246607) | 1.32 | 4.99 |  |  |  |
| hsa-miR-187 | [2685](http://genome-www4.stanford.edu/cgi-bin/SMD/source/sourceResult?choice=Gene&option=Name&criteria=2684.6111254251) | 1.31 | 3.65 |  |  |  |
| hsa-miR-425-5p | [9491](http://genome-www4.stanford.edu/cgi-bin/SMD/source/sourceResult?choice=Gene&option=Name&criteria=9490.66502739147) | 1.30 | 0 |  | BA9 | 21183010 |
| hsa-miR-487a | [11,215](http://genome-www4.stanford.edu/cgi-bin/SMD/source/sourceResult?choice=Gene&option=Name&criteria=11214.6247494922) | 1.29 | 3.65 |  | STG | 19721432 |
| hsa-miR-542-3p | [4116](http://genome-www4.stanford.edu/cgi-bin/SMD/source/sourceResult?choice=Gene&option=Name&criteria=4115.83951007813) | 1.28 | 4.99 |  |  |  |
| **hsa-miR-17-5p** | [3382](http://genome-www4.stanford.edu/cgi-bin/SMD/source/sourceResult?choice=Gene&option=Name&criteria=3382.11665229665) | 1.27 | 0 | 0.0361 | STG | 19721432 |
| hsa-miR-532 | [6998](http://genome-www4.stanford.edu/cgi-bin/SMD/source/sourceResult?choice=Gene&option=Name&criteria=6998.21008502827) | 1.26 | 0 |  |  |  |
| hsa-miR-105 | [4839](http://genome-www4.stanford.edu/cgi-bin/SMD/source/sourceResult?choice=Gene&option=Name&criteria=4838.72296394895) | 1.25 | 0 |  | BA9 | 19721432 |
| hsa-miR-199b | [3388](http://genome-www4.stanford.edu/cgi-bin/SMD/source/sourceResult?choice=Gene&option=Name&criteria=3388.33750896977) | 1.25 | 3.65 |  |  |  |
| hsa-miR-409-3p | [5040](http://genome-www4.stanford.edu/cgi-bin/SMD/source/sourceResult?choice=Gene&option=Name&criteria=5039.95354874429) | 1.24 | 3.65 |  | BA9 | 19721432 |
|  |  |  |  |  | PBMC (down) | 21727898 |
| hsa-miR-92b | [4965](http://genome-www4.stanford.edu/cgi-bin/SMD/source/sourceResult?choice=Gene&option=Name&criteria=4964.75203996986) | 1.23 | 0 |  |  |  |
| hsa-miR-150 | [13,683](http://genome-www4.stanford.edu/cgi-bin/SMD/source/sourceResult?choice=Gene&option=Name&criteria=13682.7913780862) | 1.23 | 0 |  | STG | 19721432 |
|  |  |  |  |  | PBMC (down) | 21727898 |
| hsa-miR-199a-3p | [6508](http://genome-www4.stanford.edu/cgi-bin/SMD/source/sourceResult?choice=Gene&option=Name&criteria=6507.55553496696) | 1.22 | 0 |  | STG | 19721432 |
| hsa-miR-148b | [5663](http://genome-www4.stanford.edu/cgi-bin/SMD/source/sourceResult?choice=Gene&option=Name&criteria=5662.62897724499) | 1.21 | 0 |  |  | 19721432 |
| hsa-miR-152 | [9785](http://genome-www4.stanford.edu/cgi-bin/SMD/source/sourceResult?choice=Gene&option=Name&criteria=9784.51487743779) | 1.19 | 3.65 |  | STG | 19721432 |
|  |  |  |  |  | BA9 | 21183010 |
|  |  |  |  |  | PBMC (down) | 21727898 |
| hsa-miR-495 | [11,227](http://genome-www4.stanford.edu/cgi-bin/SMD/source/sourceResult?choice=Gene&option=Name&criteria=11226.9979606314) | 1.19 | 3.65 |  |  |  |
| hsa-miR-767-5p | [4207](http://genome-www4.stanford.edu/cgi-bin/SMD/source/sourceResult?choice=Gene&option=Name&criteria=4207.21182109232) | 1.17 | 0 |  |  |  |
| **hsa-miR-134** | [10,400](http://genome-www4.stanford.edu/cgi-bin/SMD/source/sourceResult?choice=Gene&option=Name&criteria=10400.4792303465) | 1.16 | 4.99 | 0.0484 | PBMC (down) | 21727898 |
| hsa-miR-154 | [11,344](http://genome-www4.stanford.edu/cgi-bin/SMD/source/sourceResult?choice=Gene&option=Name&criteria=11343.9333686732) | 1.16 | 0 |  | STG | 19721432 |
| **hsa-miR-328** | [9110](http://genome-www4.stanford.edu/cgi-bin/SMD/source/sourceResult?choice=Gene&option=Name&criteria=9110.42426119618) | 1.16 | 3.65 | 0.0162 | STG | 19721432 |
| hsa-miR-25 | [10,958](http://genome-www4.stanford.edu/cgi-bin/SMD/source/sourceResult?choice=Gene&option=Name&criteria=10958.4479022545) | 1.11 | 0 |  |  |  |
| hsa-miR-222 | [10,393](http://genome-www4.stanford.edu/cgi-bin/SMD/source/sourceResult?choice=Gene&option=Name&criteria=10392.5608033562) | 1.11 | 3.65 |  | STG | 19721432 |
| hsa-miR-193a | [542](http://genome-www4.stanford.edu/cgi-bin/SMD/source/sourceResult?choice=Gene&option=Name&criteria=542.218730102446) | 0.68 | 0 |  | BA9 (up) | 19721432 |
| hsa-miR-512-3p | [1575](http://genome-www4.stanford.edu/cgi-bin/SMD/source/sourceResult?choice=Gene&option=Name&criteria=1575.11084837313) | 0.86 | 0 |  | BA9 (up) | 19721432 |
| hsa-miR-423 | [4464](http://genome-www4.stanford.edu/cgi-bin/SMD/source/sourceResult?choice=Gene&option=Name&criteria=4463.86390239104) | 0.87 | 0 |  | BA9 (up) | 19721432 |

*Note*: Expression analysis was performed on 37 matched pairs using Statistical Analysis of Microarrays. miRNAs with upregulated and downregulated expression are denoted in red and green, respectively. miRNAs with expression validated using qPCR are listed in bold (PMID19721432). Tissues analysed including superior temporal gyrus, peripheral blood mononucleocytes, and dorsolateral prefrontal cortex Brodmann’s Area 9 were denoted as STG, PBMC, and BA9 respectively.
