# Supplementary material for: Schizophrenia-associated MicroRNA–Gene Interactions in the Dorsolateral Prefrontal Cortex
Source: Genomics Proteomics Bioinformatics. 2020 Feb 14;17(6):623–34. doi: 10.1016/j.gpb.2019.10.003 (PMC7212302; doi:10.1016/j.gpb.2019.10.003)
Supplement: Supplementary data 3 [file mmc3.docx]

**Table S3 Expression of putatively paired gene–miRNA interactions in DLPFC (BA46) in schizophrenia**

| **miRNA** | | **Interaction confidence** | **Gene** | |
| --- | --- | --- | --- | --- |
| **ID** | **FC** |  | **ID** | **FC** |
| hsa-miR-222 | 1.107 | Experimentally observed | *PPP2R2A* | 1.089 |
| hsa-miR-17-5p | 1.267 | Experimentally observed | *CCND1* | –1.125 |
| hsa-miR-193a | –1.472 | Experimentally observed | *CCND1* | –1.125 |
| hsa-miR-105 | 1.250 | High (predicted) | *CALM2* | –1.192 |
| hsa-miR-105 | 1.250 | High (predicted) | *PEPD* | –1.112 |
| hsa-miR-105 | 1.250 | High (predicted) | *FBXO21* | 1.065 |
| hsa-miR-105 | 1.250 | High (predicted) | *SETD3* | 1.079 |
| hsa-miR-105 | 1.250 | High (predicted) | *TEX2* | 1.095 |
| hsa-miR-134 | 1.164 | High (predicted) | *PPP1R7* | –1.099 |
| hsa-miR-134 | 1.164 | High (predicted) | *SYT11* | –1.069 |
| hsa-miR-134 | 1.164 | High (predicted) | *SSBP2* | 1.100 |
| hsa-miR-148b | 1.207 | High (predicted) | *PLP1* | –1.354 |
| hsa-miR-148b | 1.207 | High (predicted) | *SCAP* | –1.138 |
| hsa-miR-148b | 1.207 | High (predicted) | *ITPK1* | –1.123 |
| hsa-miR-148b | 1.207 | High (predicted) | *GAP43* | –1.121 |
| hsa-miR-148b | 1.207 | High (predicted) | *BCL11A* | –1.108 |
| hsa-miR-148b | 1.207 | High (predicted) | *EPN2* | –1.097 |
| hsa-miR-148b | 1.207 | High (predicted) | *SELT* | –1.078 |
| hsa-miR-148b | 1.207 | High (predicted) | *PNPLA6* | –1.061 |
| hsa-miR-148b | 1.207 | High (predicted) | *RCC2* | 1.071 |
| hsa-miR-148b | 1.207 | High (predicted) | *ARHGEF12* | 1.084 |
| hsa-miR-148b | 1.207 | High (predicted) | *CANX* | 1.118 |
| hsa-miR-148b | 1.207 | High (predicted) | *CHST1* | 1.120 |
| hsa-miR-148b | 1.207 | High (predicted) | *WASL* | 1.122 |
| hsa-miR-150 | 1.230 | High (predicted) | *BASP1* | –1.090 |
| hsa-miR-150 | 1.230 | High (predicted) | *ARIH2* | 1.071 |
| hsa-miR-150 | 1.230 | High (predicted) | *FTO* | 1.092 |
| hsa-miR-17-5p | 1.267 | High (predicted) | *OLIG1* | –1.166 |
| hsa-miR-17-5p | 1.267 | High (predicted) | *PTP4A2* | –1.130 |
| hsa-miR-17-5p | 1.267 | High (predicted) | *NBL1* | –1.109 |
| hsa-miR-17-5p | 1.267 | High (predicted) | *ADAR* | –1.086 |
| hsa-miR-17-5p | 1.267 | High (predicted) | *FBXO21* | 1.065 |
| hsa-miR-17-5p | 1.267 | High (predicted) | *DNAJB9* | 1.070 |
| hsa-miR-17-5p | 1.267 | High (predicted) | *RGL1* | 1.086 |
| hsa-miR-17-5p | 1.267 | High (predicted) | *RPS6KA2* | 1.087 |
| hsa-miR-17-5p | 1.267 | High (predicted) | *MKLN1* | 1.088 |
| hsa-miR-17-5p | 1.267 | High (predicted) | *PPP2R2A* | 1.089 |
| hsa-miR-17-5p | 1.267 | High (predicted) | *TNFRSF21* | 1.090 |
| hsa-miR-17-5p | 1.267 | High (predicted) | *CAPRIN1* | 1.102 |
| hsa-miR-17-5p | 1.267 | High (predicted) | *RASL11B* | 1.194 |
| hsa-miR-193a | –1.472 | High (predicted) | *CALM2* | –1.192 |
| hsa-miR-193a | –1.472 | High (predicted) | *SFRS2* | –1.102 |
| hsa-miR-193a | –1.472 | High (predicted) | *DNAJB9* | 1.070 |
| hsa-miR-193a | –1.472 | High (predicted) | *ARHGEF12* | 1.084 |
| hsa-miR-193a | –1.472 | High (predicted) | *RGL1* | 1.086 |
| hsa-miR-193a | –1.472 | High (predicted) | *MKLN1* | 1.088 |
| hsa-miR-193a | –1.472 | High (predicted) | *TNFRSF21* | 1.090 |
| hsa-miR-193a | –1.472 | High (predicted) | *CAPRIN1* | 1.102 |
| hsa-miR-199a-3p | 1.222 | High (predicted) | *PHYHIPL* | –1.155 |
| hsa-miR-199a-3p | 1.222 | High (predicted) | *ITPK1* | –1.123 |
| hsa-miR-199a-3p | 1.222 | High (predicted) | *PPP2R2A* | 1.089 |
| hsa-miR-199b | 1.247 | High (predicted) | *PIK4CA* | –1.121 |
| hsa-miR-199b | 1.247 | High (predicted) | *PNPLA6* | –1.061 |
| hsa-miR-199b | 1.247 | High (predicted) | *ARIH2* | 1.071 |
| hsa-miR-199b | 1.247 | High (predicted) | *ARHGEF12* | 1.084 |
| hsa-miR-199b | 1.247 | High (predicted) | *CAPRIN1* | 1.102 |
| hsa-miR-199b | 1.247 | High (predicted) | *MBP* | 1.157 |
| hsa-miR-222 | 1.107 | High (predicted) | *SFRS2* | –1.102 |
| hsa-miR-222 | 1.107 | High (predicted) | *PLEKHA2* | 1.109 |
| hsa-miR-328 | 1.156 | High (predicted) | *KIAA1715* | 1.150 |
| hsa-miR-382 | 1.475 | High (predicted) | *ARIH2* | 1.071 |
| hsa-miR-409-3p | 1.239 | High (predicted) | *SELT* | –1.078 |
| hsa-miR-409-3p | 1.239 | High (predicted) | *DNAJB9* | 1.070 |
| hsa-miR-409-3p | 1.239 | High (predicted) | *RAB10* | 1.108 |
| hsa-miR-425-5p | 1.299 | High (predicted) | *CALM2* | –1.192 |
| hsa-miR-425-5p | 1.299 | High (predicted) | *CCND1* | –1.125 |
| hsa-miR-425-5p | 1.299 | High (predicted) | *EPN2* | –1.097 |
| hsa-miR-433 | 1.348 | High (predicted) | *EPN2* | -1.097 |
| hsa-miR-433 | 1.348 | High (predicted) | *GRSF1* | 1.095 |
| hsa-miR-433 | 1.348 | High (predicted) | *LSM14A* | 1.098 |
| hsa-miR-433 | 1.348 | High (predicted) | *MBP* | 1.157 |
| hsa-miR-495 | 1.188 | High (predicted) | *ETV5* | –1.187 |
| hsa-miR-495 | 1.188 | High (predicted) | *PCDH17* | –1.153 |
| hsa-miR-495 | 1.188 | High (predicted) | *CCND1* | –1.125 |
| hsa-miR-495 | 1.188 | High (predicted) | *PPP2R2C* | –1.114 |
| hsa-miR-495 | 1.188 | High (predicted) | *BCL11A* | –1.108 |
| hsa-miR-495 | 1.188 | High (predicted) | *MKLN1* | 1.088 |
| hsa-miR-495 | 1.188 | High (predicted) | *TNFRSF21* | 1.090 |
| hsa-miR-495 | 1.188 | High (predicted) | *FOXK1* | 1.093 |
| hsa-miR-495 | 1.188 | High (predicted) | *GRSF1* | 1.095 |
| hsa-miR-495 | 1.188 | High (predicted) | *PLEKHA2* | 1.109 |
| hsa-miR-495 | 1.188 | High (predicted) | *CANX* | 1.118 |
| hsa-miR-502 | 2.763 | High (predicted) | *CCR6* | –1.110 |
| hsa-miR-502 | 2.763 | High (predicted) | *XAB1* | 1.135 |
| hsa-miR-512-3p | –1.161 | High (predicted) | *MKLN1* | 1.088 |
| hsa-miR-512-3p | –1.161 | High (predicted) | *WASL* | 1.122 |
| hsa-miR-519c | 1.514 | High (predicted) | *TEX2* | 1.095 |
| hsa-miR-519c | 1.514 | High (predicted) | *KIAA1715* | 1.150 |
| hsa-miR-542-3p | 1.279 | High (predicted) | *GRSF1* | 1.095 |
| hsa-miR-542-3p | 1.279 | High (predicted) | *CAPRIN1* | 1.102 |
| hsa-miR-590 | 1.604 | High (predicted) | *BCL11A* | –1.108 |
| hsa-miR-590 | 1.604 | High (predicted) | *ARHGEF12* | 1.084 |
| hsa-miR-767-5p | 1.169 | High (predicted) | *BASP1* | –1.090 |
| hsa-miR-767-5p | 1.169 | High (predicted) | *CACNA2D3* | 1.121 |
| hsa-miR-92b | 1.231 | High (predicted) | *CALM2* | –1.192 |
| hsa-miR-92b | 1.231 | High (predicted) | *CXXC5* | –1.156 |
| hsa-miR-92b | 1.231 | High (predicted) | *LIMCH1* | –1.139 |
| hsa-miR-92b | 1.231 | High (predicted) | *GAP43* | –1.121 |
| hsa-miR-92b | 1.231 | High (predicted) | *BCL11A* | –1.108 |
| hsa-miR-92b | 1.231 | High (predicted) | *SELT* | –1.078 |
| hsa-miR-92b | 1.231 | High (predicted) | *DNAJB9* | 1.070 |
| hsa-miR-92b | 1.231 | High (predicted) | *NUDCD3* | 1.086 |
| hsa-miR-92b | 1.231 | High (predicted) | *RGL1* | 1.086 |
| hsa-miR-92b | 1.231 | High (predicted) | *DCLK2* | 1.094 |
| hsa-miR-92b | 1.231 | High (predicted) | *TEX2* | 1.095 |
| hsa-miR-92b | 1.231 | High (predicted) | *ELFN2* | 1.109 |
| hsa-miR-92b | 1.231 | High (predicted) | *TAF15* | 1.111 |
| hsa-miR-92b | 1.231 | High (predicted) | *CHST1* | 1.120 |
| hsa-miR-92b | 1.231 | High (predicted) | *WASL* | 1.122 |

*Note*: Ingenuity Pathways Analysis with microRNA Target Filter was paired by fold change. Upregulated and downregulated genes/miRNAs are put in red and green, respectively. Only high-confidence predictions or experimentally validated interactions (according to IPA Knowledge Base) were included.
