# Supplementary material for: Schizophrenia-associated MicroRNA–Gene Interactions in the Dorsolateral Prefrontal Cortex
Source: Genomics Proteomics Bioinformatics. 2020 Feb 14;17(6):623–34. doi: 10.1016/j.gpb.2019.10.003 (PMC7212302; doi:10.1016/j.gpb.2019.10.003)
Supplement: Supplementary data 6 [file mmc6.docx]

**Table S6 Cohort demographics and tissue characterisation**

| **Pair** | **Diag** | **Sex** | **Age** | **pH** | **PMI** | **Hemi** | **Cause of death** | **Toxicology** | **APD** | **CPE** | **AO** | **DOI** |
| --- | --- | --- | --- | --- | --- | --- | --- | --- | --- | --- | --- | --- |
| 1 | CRS | F | 51 | 5.7 | 12 | R | Li toxicity & congestive cardiac failure | Li: 20 mg/L (fatal); midazolam: 0.02 mg/ L | T | 570 | 35 | 16 |
| 2 | CDS | M | 56 | 6.2 | 15 | L | Chronic airways disease | NAD | TO | 450 | 21 | 35 |
| 3 | CUS | M | 52 | 6.4 | 46 | R | Cardiomegaly (cardiomyopathy) | N/A | A | 500 | 19 | 33 |
| 4 | CRS | M | 51 | 6.5 | 21 | L | Ischaemic heart disease | Thioridazine: 2.2 mg/ L (fatal); mesoridazine: 2.4 mg/ L (fatal) | TO | - | 27 | 24 |
| 5 | CPS | M | 54 | 6.2 | 27.5 | R | Coronary artery thrombosis | Chlorpromazine: 0.7 mg/L; diazepam: < 0.1 mg/L; nordiazepam: 0.1; insulin: 2 uU/mL | T | 1560 | 19 | 35 |
| 6 | CPS | F | 58 | 6.3 | 19 | R | Sepsis & chronic renal failure | Morphine: 0.06 mg/L; codeine: 0.05 mg/L; carbamazepine: 7 mg/L; pethidine: 0.1 mg/L; paracetamol: 6 mg/L; metoclopramide: 0.1 mg/L; diazepam: < 0.1 mg/L | T | 450 | 19 | 39 |
| 7 | CPS | F | 66 | 6.3 | 12.5 | R | Faecaloid peritonitis | N/a | T | - | 19 | 47 |
| 8 | CUS | F | 55 | 6.3 | 33.5 | L | AMS toxicity & coronary artery disease | Amisulpride: 18 mg/L; clozapine: 1.4 mg/L | T | 1300 | 17 | 38 |
| 9 | CPS | M | 55 | 6.4 | 72 | R | Ischaemic heart disease | Metoprolol: 0.2 mg/L | TO | - | 30 | 26 |
| 10 | CPS | F | 54 | 6.4 | 29 | R | Asthma | Citalopram: 0.6 mg/L | T | 700 | 19 | 35 |
| 11 | CDS | F | 67 | 6.4 | 27 | L | Empyema | Benztropine: 0.2 mg/L; mesoridazine: 0.9 mg/L; thioridazine: 0.6 mg/L; paracetamol: < 3.0 mg/L | TO | 350 | 21 | 46 |
| 12 | CPS | M | 75 | 6.6 | 36 | L | Ischaemic heart disease | Olanzapine: 0.2 mg/L; fluvoxamine: 0.7 mg/L | T | 500 | <33 | 44 |
| 13 | CPS | M | 40 | 6.5 | 21.5 | L | Dihydrocodeine toxicity & sleep apnoea | Valproic acid: 20 mg/L; dihydrocodeine 0.7 mg/L; quetiapine 0.3mg/L; sertraline 0.3 mg/L | T | 986 | 17 | 23 |
| 14 | CUS | M | 44 | 6.5 | 27-43.5 | L | Hanging | Urine THC detected | T | 500 | 27 | 17 |
| 15 | CUS | M | 27 | 6.6 | 10 | L | Clozapine toxicity | Clozapine: 8.6 mg/L (fatal) | T | 300 | 18 | 11 |
| 16 | CPS | M | 51 | 6.7 | 18 | L | Ischaemic heart disease | N/A | T | 150 | 21 | 30 |
| 17 | CPS | M | 52 | 6.7 | 8.35 | R | Ischaemic heart disease | Temazepam: < 0.1 mg/L | T | 680 | 21 | 31 |
| 18 | CPS | M | 33 | 6.8 | 48 | L | Hanging | Doxylamine: 0.9 mg/L; olanzapine: 0.2 mg/L; paracetamol: 3 mg/L | A | 250 | 22 | 12 |
| 19 | CPS | M | 30 | 6.8 | 24 | L | CO poisoning - suicide | CO: 74% saturation; clozapine: 0.7 mg/L; HIV | A/T | 300 | 26 | 3.5 |
| 20 | CDS | F | 56 | 6.8 | 34 | L | Pulmonary thrombo-embolism | Paracetamol: 4.5 mg/L | TO | 760 | 17 | 40 |
| 21 | CUS | M | 27 | 6.8 | 38.5 | L | Myocarditis | Clozapine: 0.9 mg/L | A | 200 | 23 | 4 |
| 22 | CPS | M | 57 | 7 | 33-38 | L | Cardiac arrythmia | Thioridazine: 0.6 mg/L; seraline: < 0.1 mg/L | T | 700 | 30 | 26 |
| 23 | CPS | M | 57 | 6.7 | 48 | R | Atherosclerotic cardiovascular disease | Carbamazepine: 10 mg/L; citalopram: 0.2 mg/L; quetiapine: < 0.1 mg/L | T | 618 | 40 | 17 |
| 24 | CPS | M | 27 | 6.8 | 33 | R | Hanging | Negative | T | - | 19 | 9 |
| 25 | CDS | M | 59 | 6.9 | 26.5 | R | Unknown | N/A | T | 750 | 21 | 39 |
| 26 | CUS | M | 67 | 6.8 | 5 | L | Ischaemic heart disease | N/A | TO | 1340 | 26 | 41 |
| 27 | CPS | F | 61 | 6.9 | 42 | R | Ischaemic heart disease | Clozapine: 1.1 mg/L; diazepam: 0.2 mg/L; audanosine: 0.4 mg/L; nordiazepame: 0.4 mg/L; olanzapine: 0.2 mg/L | TO | 1200 | 19 | 42 |
| 28 | CDS | M | 32 | 7 | 26 | L | Hanging | Negative | T | 190 | 19 | 13 |
| 29 | CPS | F | 56 | 7.1 | 39 | R | Undetermined (obesity / hepatic fatty changes) | Thioridazine: 1 mg/L; mesoridazine: 0.6 mg/L | T | 580 | 24 | 32 |
| 30 | CUS | F | 68 | 6.2 | 32 | L | Acute pancreatitis | EtOH: 0.55 g/L; paracetamol: 3 mg/L | T | 190 | 23 | 46 |
| 31 | BPT | M | 34 | 7 | 26 | R | Hanging | Carbamazepine: 1 mg/L | A | 250 | 27 | 8 |
| 32 | CDPS | F | 33 | 6.9 | 50 | R | Hanging | Negative | AO | 95 | 14 | 19 |
| 33 | BPT | M | 57 | 6.4 | 28 | R | Chronic obstructive airway disease | Paracetamol: 3 mg/L; Li: 4.6 mg/L | T | 415 | 30 | 27 |
| 34 | BPT | F | 61 | 6.4 | 17 | R | Myocarditis | Clozapine: 0.4 mg/L; Li: 0.1 mg/L; nil EtOH | T | 100 | 31 | 30 |
| 35 | CDPS | M | 30 | 7 | 26 | L | Hanging | Venlafaxine: 0.9 mg/L | A | 285 | 27 | 4 |
| 36 | CDPS | F | 73 | 6.9 | 17-19 | L | Right ventricular dysplasia | Doxepin: 0.1 mg/L; chlorpheniramine: 0.01 mg/L; trifluoperazine: 0.01 mg/L; Codeine: 0.05 mg/L; pseudoephedrine: 0.1 mg/L; paracetamol: 10 mg/L | TO | 300 | 36 | 37 |
| 37 | CDPS | M | 73 | 6.8 | 14 | L | Asphyxia | Fluoxetine: 0.2 mg/L; 7-amino nitrazepam: 0.1 mg/L; diazepan: < 0.1 mg/L; paracetamol: < 3 mg/L | T | 380 | 21 | 50 |
| 1 | CTR | M | 46 | 5.8 | 29 | L | Acute myocardial infarction | N/A |  |  |  |  |
| 2 | CTR | M | 60 | 6 | 25 | R | Bacterial peritonitis; ascites; carcinomatosis | Negative |  |  |  |  |
| 3 | CTR | M | 37 | 6.2 | 11 | R | Pulmonary embolism | Negative |  |  |  |  |
| 4 | CTR | M | 56 | 6.6 | 24 | R | Coronary artery atheroma | N/A |  |  |  |  |
| 5 | CTR | M | 61 | 6.3 | 27.5 | R | Unknown | N/A |  |  |  |  |
| 6 | CTR | M | 74 | 6.3 | 10 | L | Respiratory arrest | N/A |  |  |  |  |
| 7 | CTR | F | 78 | 6.4 | 11 | R | Pulmonary fibrosis | N/A |  |  |  |  |
| 8 | CTR | F | 56 | 6.5 | 23 | R | Massive pulmonary thrombo-embolus | N/A |  |  |  |  |
| 9 | CTR | F | 60 | 6.5 | 21 | L | Ischaemic heart disease | EtOH: 2.51 g/L; paracetamol < 3 mg/L |  |  |  |  |
| 10 | CTR | M | 60 | 6.6 | 13 | L | Acute myocardial infarction | Negative |  |  |  |  |
| 11 | CTR | M | 58 | 6.6 | 12 | L | Ischaemic heart disease | N/A |  |  |  |  |
| 12 | CTR | M | 73 | 6.6 | 48 | R | Ischaemic heart disease | N/A |  |  |  |  |
| 13 | CTR | M | 46 | 6.7 | 25 | R | Mitral valve prolapse | Negative |  |  |  |  |
| 14 | CTR | F | 49 | 6.6 | 15 | R | Arrhytmogenic right venticular dysplasia | Chloride ion: 118 mM |  |  |  |  |
| 15 | CTR | M | 34 | 6.5 | 20.5 | R | Acute exacerbation of asthma | N/A |  |  |  |  |
| 16 | CTR | M | 44 | 6.7 | 50 | L | Ischaemic heart disease | Negative |  |  |  |  |
| 17 | CTR | M | 50 | 6.6 | 19 | L | Ischaemic heart disease | NAD |  |  |  |  |
| 18 | CTR | M | 43 | 6.7 | 13 | R | Thrombotic coronary artery occlusion | Negative |  |  |  |  |
| 19 | CTR | M | 38 | 6.7 | 13.5 | L | Atherosclerotic cardiovascular disease | N/A |  |  |  |  |
| 20 | CTR | M | 54 | 6.8 | 29 | R | Coronary artery atheroma | Negative |  |  |  |  |
| 21 | CTR | M | 18 | 6.8 | 33 | L | Probable hypertrophic cardiomyopathy | Paracetamol: 24 mg/L; lignocaine: 1 mg/L |  |  |  |  |
| 22 | CTR | F | 51 | 7.2 | 37.5 | R | Acute myocardial infarction | N/A |  |  |  |  |
| 23 | CTR | M | 53 | 6.7 | 27 | R | Acute myocardial infarct | N/A |  |  |  |  |
| 24 | CTR | F | 33 | 6.9 | 24 | L | Cardiac arrythmia; myocardial fibrosis | EtOH not detected |  |  |  |  |
| 25 | CTR | M | 59 | 7 | 20 | R | Coronary thrombosis | N/A |  |  |  |  |
| 26 | CTR | M | 56 | 7 | 37 | R | Hypertension & cardiomegaly | N/A |  |  |  |  |
| 27 | CTR | M | 57 | 6.9 | 18 | L | Ischaemic heart disease | HIV negative |  |  |  |  |
| 28 | CTR | M | 37 | 6.9 | 24 | R | Electrocution | Codeine: < 0.5 mg/L; paracetamol: 3 mg/L; lignocaine: < 0.5 mg/L |  |  |  |  |
| 29 | CTR | M | 55 | 7.2 | 20 | L | Cardiac arrest | N/A |  |  |  |  |
| 30 | CTR | M | 78 | 6.3 | 6.5 | L | Dehydration; adenocarcinoma | N/A |  |  |  |  |
| 31 | CTR | M | 37 | 6.8 | 21 | L | Ischaemic heart disease | N/A |  |  |  |  |
| 32 | CTR | F | 21 | 6.8 | 39.5 | R | Primary cardiac arrhythmia | N/A |  |  |  |  |
| 33 | CTR | M | 62 | 6.6 | 37.5 | R | Acute myocardial infarction | Negative |  |  |  |  |
| 34 | CTR | M | 50 | 6.7 | 29 | R | Ischaemic heart disease | Negative |  |  |  |  |
| 35 | CTR | M | 24 | 7 | 43 | R | Idiopathic cardiac arrhythmia | Negative |  |  |  |  |
| 36 | CTR | M | 64 | 7 | 39.5 | R | Coronary artery thrombosis | Negative |  |  |  |  |
| 37 | CTR | M | 60 | 7 | 21.5 | R | Ischaemic heart disease | N/A |  |  |  |  |

*Note*: Diag, diagnosis; CRS, chronic residual schizophrenia; CDS, chronic differential schizophrenia; CUS, chronic undifferentiated schizophrenia; CPS, chronic paranoid schizophrenia; BPT, bipolar type; CDPS, chronic depressive schizophrenia; CTR, control; PMI, post mortem interval (hours); Hemi, brain hemisphere; APD, antipsychotic drug class (A, predominantly atypical; T, predominantly typical; TO, typical only; A/T, equal); CPE; chloropromazine equivalent (mg/day); AO, age of onset; DOI, duration of illness (years).
